# Spanish versión of The App Behavior Change Scale (ABACUS-Es): Adaptation and validation study

**DOI:** 10.1371/journal.pone.0314753

**Published:** 2024-12-05

**Authors:** Ruben Martin-Payo, Covadonga González-Nuevo-Vázquez, Estela Álvarez-Gómez, Shelini Surendran, Judit Cachero-Rodríguez, María del Mar Fernandez-Alvarez

**Affiliations:** 1 Facultad de Medicina y Ciencias de la Salud, Universidad de Oviedo, Oviedo, Asturias, Spain; 2 PRECAM Research Group, Instituto de Investigación Sanitaria del Principado de Asturias, Oviedo, Asturias, Spain; 3 Facultad de Psicología, Universidad de Oviedo, Oviedo, Asturias, Spain; 4 Escuela de Enfermería Fundación Jimenez Díaz, Madrid, Spain; 5 School of Biosciences and Medicine, Faculty of Health and Medical Sciences, University of Surrey, Guildford, United Kingdom; Northumbria University, UNITED KINGDOM OF GREAT BRITAIN AND NORTHERN IRELAND

## Abstract

**Background:**

Health applications have grown exponentially in the last decade and are well accepted by users and health services. It is essential that digital tools meet the needs of the target users and are reliable, hence the importance of evaluating them. The aim of this study was to adapt The App Behavior Change Scale (ABACUS) into the Spanish language and to assess their metric properties.

**Methods:**

This was an study undertaken in two phases: (1) translation and cultural adaptation process; (2) psychometric evaluation. 65 physical activity apps were included in the study (9 running Apple, 50 running Android and 6 running both). Cross-cultural adaptation, translation, and metric evaluation was made. The internal consistency and test-retest reliability of the Spanish version of the ABACUS was evaluated using physical activity apps. Two reviewers independently rated the apps using the translated scale. The apps were then rated again 2 weeks later by the same reviewers to measure test-retest reliability.

**Results:**

No major differences were observed between both versions of ABACUS (original and Spanish). Discrimination indices (item-scale correlation) obtained appropriate results for both raters. ABACUS-Es presented an excellent interrater reliability (0.82, 95% CI 0.80–0.84) and excellent internal consistency (Cronbach alpha = 0.82).

**Conclusions:**

The Spanish version of ABACUS, ABACUS-Es, is an instrument with adequate metric properties to measure the potential behavior change of apps from health professionals and app developers. ABACUS will help guide future research efforts in the development and evaluation of apps to promote health behaviors, and provides guidance for app developers to include adequate behavior change techniques in apps in Spanish to be used to promote health behaviors.

## Introduction

EHealth is conceptualized as “*the use of internet technology by the public*, *health workers*, *and others to access health and lifestyle information*, *services and support*” [1]. As part of eHealth, it is important to consider the use of mobile health (mHealth), including mobile apps and other wearable devices for health monitoring [2]. Health apps have exponentially grown in the last decade and are well accepted by users and healthcare services [3, 4]. Digital interventions based on app usage have generally showed to be effective in terms of costs and health outcomes [5, 6]. A high percentage of these apps are offered in digital markets with limited consideration of relevant aspects, such as its content or quality aspects [7, 8]. Consequently, it is important to highlight that an inadequate app design could cause harm to users [9].

What has been described above suggests the formulation of the following question: Can any app related to e-health be recommended to any person? The answer seems to be clear “no”. Not all apps can be recommended for any patients, as there must be consideration of their personal characteristics [10–12].

As suggested in the framework designed by Norgaard et al. [13], it is essential that digital tools cater the needs of the target users. For example, if a health professional wants to recommend apps to promote a healthy diet during pregnancy, it seems reasonable to recommend apps specifically designed to this aim [14], rather than those designed for adults in general [15]. To illustrate this, mention can be made of the results of the study carried out by Muñoz-Mancisidor et al. [12] regarding the total apps found in digital markets for recommendation during pregnancy. Only 5.6% of them could be recommended on the basis of their content, quality and strategies for behavior change. This undoubtedly poses a challenge for healthcare professionals, who must become familiarized with evaluations of this kind, in order to be able to recommend the app best suited for their patients.

Additionally, other app characteristics need to be considered, including its quality [8]. There are some tools specifically designed to assess the quality of the apps. A widely used tool for health app quality evaluation from the health professional and user perspective, are the Mobile Application Rating Scale and the User Mobile Application Rating Scale respectively [16, 17]. Both was initially designed in English and subsequently adapted into Spanish characteristics [18, 19].

Finally, the inclusion of behavior change techniques is crucial in apps designed to promote healthy behaviors. Some authors suggest that it is essential to use a combination of behavior change techniques and behavior change theory to increase intervention effectiveness [20]. In a similar way, other authors who conclude that the potential of apps to promote health-related behaviors could be enhanced by including behavior change techniques in their design [21]. As an example, and taking cardiovascular disease as a reference, representing the leading cause of mortality worldwide [22], it is well known that adopting healthy behaviors could contribute to reduce the risk of such disease in the general population [23], for example by using apps [24].

Two behavior change taxonomies can be highlighted. The Behavior change technique Taxonomy v1, developed by Michie et al. [25], is a consensus-based taxonomy of 93 behavior change techniques for reporting intervention content. ABACUS, developed by McKay et al. [26], is a 21-item scale that can be used to determine the behavior change potential specifically of apps, showing adequate internal coherence (Cronbach alpha = 0.93). This tool has been used to evaluate apps related to different aspects of health, including neurological disorders [27], vaping cessation [28], low back pain [29], well-being [21], physical activity [30], and physiotherapy care [31]. Likewise, scientific literature has been found that contemplates its use to evaluate apps in languages other than English, such as Dutch [32] or Russian [33]. However, no adaptations of ABACUS to these languages have been identified, and the authors have used the original version, which suggests adequate proficiency in both languages.

Adapting the tools to the native language of the evaluators widens the margins for use, in the sense that they do not need to speak the language of the tool (English in the case of ABACUS), and reduces the probability of information bias derived from imprecise measurements.

Since there was no found a taxonomy like those in Spanish, we developed this study with the aim to adapt ABACUS into the Spanish language and to assess their metric properties. The present study attempts to offer ABACUS in Spanish, to be used by developers, researchers and healthcare professionals in the development or recommendation of app for health purposes. This will contribute to reliably evaluate the behavior change strategies included in the apps as a criterion recommended in the scientific literature for indicating the effectiveness of behavior change. On the other hand, it eliminates the barrier related to the need to speak English for adequate use of ABACUS by developers, researchers and healthcare professionals.

## Methods

### Ethics

Ethical approval was obtained from the Ethics Committee of the Principado de Asturias (reference 2021.569).

### Participants (Unit of analysis: Apps)

Apps available in the “Health and fitness” category on iTunes, Google Play, or both stores in the period between June to September 2022 were included. The following inclusion criteria were applied: i) apps targeting adults, ii) focusing on physical activity or exercise, iii) free to use, iv) available in Spanish. The exclusion criteria were the following: i) focusing on non-physical activity-based interventions; ii) requiring a wearable device to be used.

The assessment process consisted of four phases: i) app identification using the search term “physical activity”; ii) initial screening based on the apps marketing information; iii) downloading apps to an Apple device (iPad pro), an Android device (Samsung Galaxy A53); iv) removing duplicates from the Apple and/or Android device based on the criteria of the reviewer; and v) application of the inclusion and exclusion criteria commented above. Two reviewers independently assessed the behavior change techniques included in the apps using ABACUS.

According to Zou [34], a minimum sample size of 41 is required to determine whether the true inter-rater reliability lies within 0.15 of a sample observation of 0.80, with 87% assurance.

### Instruments

The original version of ABACUS was cross-culturally adapted for the Spanish language. Consequently, a cross-cultural adaptation, translation, and measure of metric properties were developed. The corresponding STROBE checklist was used in this study.

The ABACUS scale [26] was designed to measure the potential behavior change of apps. It consists of 21 items grouped into 4 categories: knowledge and information (5 items), goals and planning (3 items), feedback and monitoring (7 items), and actions (6 items). Each item represents a behavior change technique, and its inclusion in the app is rated dichotomously (behavior change technique not included = 0 or included = 1).

### Procedure

The adaptation and translation processes were developed using the method proposed by Ramada-Rodilla et al. [35] Initially, a translation into Spanish was independently made by two native Spanish speakers proficient in the English language. Subsequently, both created a consensus version after assessing both translations together. An expertise panel composed by both translators, a nurse expert in behavioral change interventions, an expert in methodology and expert in Spanish language. This version was back translated into English and the original author checked the semantic equivalence [21]. Finally, the metric properties were assessed: reliability, internal consistency and test-retest reliability of the scale.

### Data analysis

The ABACUS-Es was used by two raters to review 65 physical activity apps.

Reliability of the scale was assessed using the Cohen kappa coefficient. Consistent with previous research [36], a kappa between 0.01 and 0.20 indicates slight agreement, between 0.21 and 0.40 fair, between 0.41 and 0.60 moderate, between 0.61 and 0.80 substantial and between 0.81 and 1.00 almost perfect. However, a negative kappa indicates less agreement than that would be expected by chance and suggests that there may have been inconsistencies in how measures were applied.

The internal consistency of the scale was calculated using Cronbach alpha. Interrater reliability was determined by interclass coefficient (ICC). Percentage agreement was also calculated.

The test-retest reliability of the scores assigned by the raters with the 15 apps was obtained by calculating the Pearson correlations between the two points in time.

All analyses were conducted using SPSS for Windows, version 27.0 (SPSS Inc).

## Results

467 apps were identified. 345 were eliminated, because either their final objective was not physical activity, they included aspects related to meditation or mindfulness, they did not consider physical activity as a whole but instead focused on specific muscle groups, they involved economic transactions, or they required the use of an additional or wearable device.

**Fig 1:**
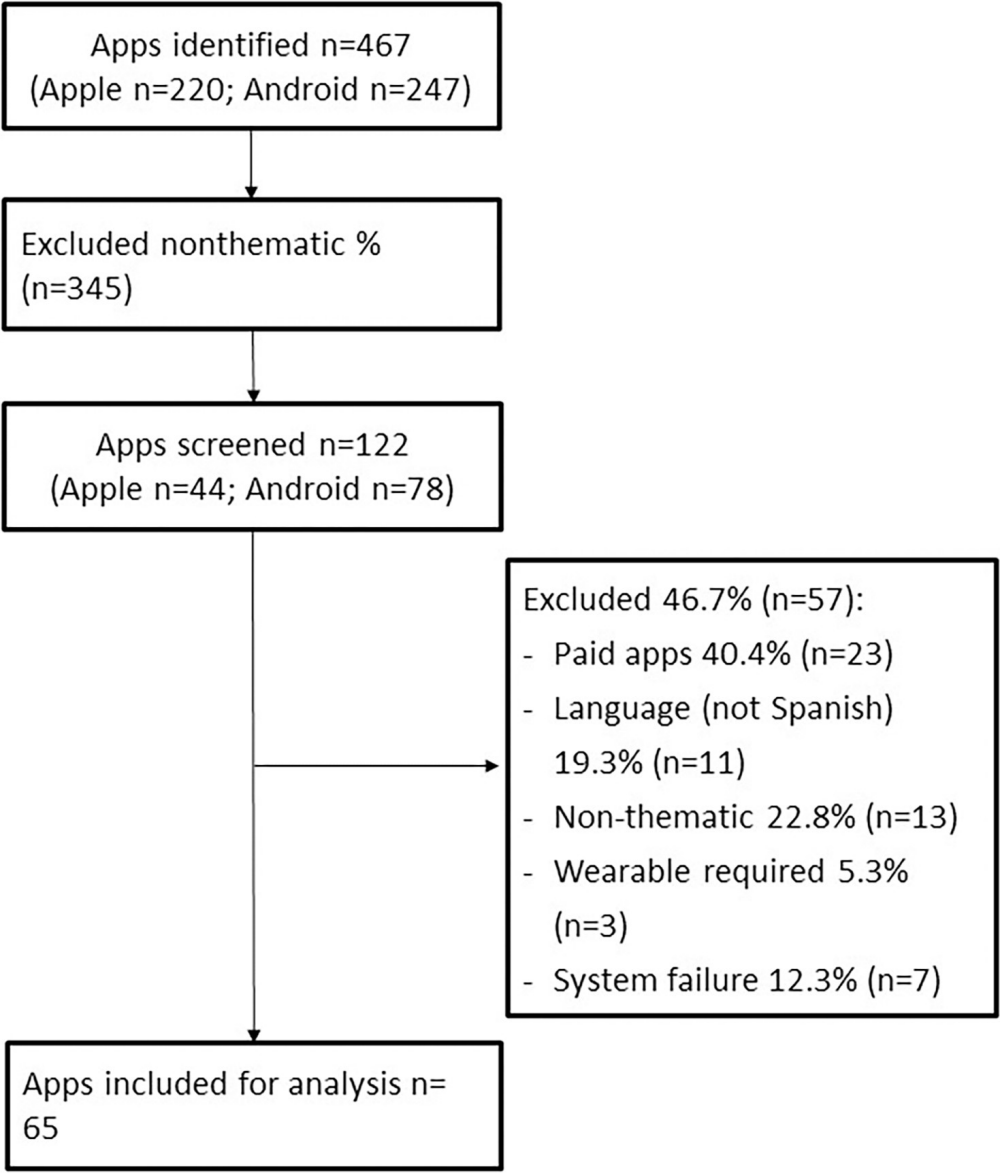
Flowchart of the app search process.

Subsequently, apps were downloaded for screening and a second review was carried out. After downloading the apps, a further 57 apps were eliminated because: i) paid apps (40.4%); ii) not available in Spanish (19.3%); iii) non focusing on physical activity (22.8%); iv) requiring a wearable (5.3%); v) malfunctioning or not working (12.3%) (Fig 1). 65 apps were included in the study (9 running Apple, 50 running Android and 6 running both).

### Cross-cultural adaptation and translation process

Following the methodology described in the “cross-cultural adaptation and translation process” section. No major differences were observed between the two independent translations, and no major modifications of the specific terminology were required. Therefore, the final Spanish version of ABACUS was created upon reaching consensus on any discrepancies; both the conceptual analysis and the translation were considered relevant and appropriate for the Spanish culture. Additionally, the original author approved the version after checking the semantic equivalence.

### Metric properties assessment

The average score of ABACUS-Es was 8.8 (SD = 2.99), which ranged from 2 to 17. Reliability analysis testing was conducted with the 65 physical activity apps downloaded from the app store. All apps were rated independently by two reviewers. It was found that over 70% of the questions had high percentage agreement among reviewers (over 80%), with the scale overall reporting excellent interrater reliability (2-way mixed ICC = 0.82, 95% CI 0.80–0.84) and excellent internal consistency (Cronbach alpha = 0.82).

Although four questions returned a negative kappa, most of the items returned an agreement above moderate (Table 1).

**Table 1:** Percentage agreement and reliability of App Behavior Change Scale (n = 65 apps).

		Kappa	CI lower (95%)	CI upper (95%)	% agreement
Category 1	Knowledge and information				
Item 1.1	Does the app have the ability to customize and personalize some features?	0.57	0.32	0.81	84.62
Item 1.2	Was the app created with expertise and/or does the app provide information that is consistent with national guidelines?	0.44	0.23	0.64	84.62
Item 1.3	Does the app ask for baseline information?	0.67	0.43	0.91	86.15
Item 1.4	Does the app provide instruction on how to perform the behavior?	0.49	0.20	0.79	73.85
Item 1.5	Does the app provide information about the consequences of continuing and/or discontinuing behavior?	-0.03	-0.19	0.14	89.06
Category 2	Goals and planning				
Item 2.1	Does the app ask for willingness for behavior change?	0.33	0.09	0.56	86.15
Item 2.2	Does the app allow for the setting of goals?	0.58	0.34	0.82	81.54
Item 2.3	Does the app have the ability to review goals, update, and change when necessary?	0.60	0.35	0.84	81.54
Category 3	Feedback and monitoring				
Item 3.1	Does the app give the user the ability to quickly and easily understand the difference between current action and future goals?	0.33	0.14	0.53	67.69
Item 3.2	Does the app have the ability to allow the user to easily selfmonitor behavior?	0.59	0.36	0.83	87.69
Item 3.3	Does the app have the ability to share behaviors with others (including social media or forums) and/or allow for social comparison?	0.32	0.09	0.56	69.23
Item 3.4	Does the app have the ability to give the user feedback—either from a person or automatically?	0.59	0.35	0.82	89.23
Item 3.5	Does the app have the ability to export data from app?	0.66	0.42	0.89	65.63
Item 3.6	Does the app provide a material or social reward or incentive?	0.64	0.40	0.88	80.00
Item 3.7	Does the app provide general encouragement?	0.33	0.09	0.57	67.69
Category 4	Actions				
Item 4.1	Does the app have reminders and/or prompts or cues for activity?	0.47	0.24	0.70	78.46
Item 4.2	Does the app encourage positive habit formation?	0.50	0.30	0.79	96.23
Item 4.3	Does the app allow or encourage for practice or rehearsal, in addition to daily activities?	-0.04	-0.28	0.20	92.31
Item 4.4	Does the app provide opportunity to plan for barriers?	1			100
Item 4.5	Does the app assist with or suggest restructuring the physical or social environment?	-0.02	-0.30	0.21	96.88
Item 4.6	Does the app assists with distraction or avoidance?	1			100

Finally, the test-retest reliability in the first rater was r = 0.99 and for the second rater was r = 0.94.

## Discussion

This study confirms the metric properties of the Spanish version of ABACUS (ABACUS-Es), a new instrument in Spanish language that measures the potential behavior change of apps. The conceptual and semantic equivalences suggests that the Spanish version is reliable in fulfilling the same purpose of the original scale and that it is functionally equivalent to the original version [26].

Similar metric properties were obtained by the present study as those presented in the English version of the scale [26]. Both instruments have excellent percentage agreement among reviewers (>80%), inter-rater reliability (0.92) and internal consistency. ABACUS-Es presented a slightly lower internal consistency (0.82) than the English version (0.92) [26]. Lower agreement among reviewers were found in category 3. Since there is no hypothesis to explain differences, further studies should be developed to explain the observed data.

The average score was 8.8 (SD = 2.99), which ranged from 2 to 17. This result is consistent with previous research where similar scores were found. Previous research that includes apps focused on smoking, alcohol use, physical activity, nutrition, and mental well-being scores for ABACUS ranged from 1 to 17, out of 21, with an average score of 7.8 (SD 2.8) [37]. In a research focusing on health apps for patients with a chronic condition or multimorbidity the average score was 8.07 (SD 2.30) [37].

ABACUS has been used to evaluate the behavior change potential of digital tools [14, 38, 39] or as a theory-driven approach to design mHealth interventions [40, 41]. So, it is supposed that ABACUS-Es, in combination with the content and quality assessment, will be use in a similar way. For example, to guide Spanish health professionals about which app would be best to recommend to their patients. As previous research concludes, identifying behavior change techniques that are most effective in promoting and maintaining positive health behaviors is essential to consider in app development because they significantly improve health behaviors and outcomes [42]. Thus, based on this premise, identifying behavior change techniques in the developed apps, also could contribute to improved health behaviors.

Previous authors found mixed effectiveness in app-based interventions, for example, same behavior change techniques have different effects depending on personal or clinical characteristics of the targe groups [43] or the health topic addressed [44]. It suggests the need to identify and use adequate behavior change techniques for obtain the best outcomes [3, 45, 46]. The none existence of a specific tool to perform this identification, could be considered as a barrier. Thus, it is important to highlight that the Spanish version of ABACUS contributes to providing a standardized method of evaluating smartphone apps for behavior change.

The issue addressed arose from the knowledge that a considerable percentage of health apps offered in digital markets have limited consideration of relevant aspects, such as the behavior change techniques included. Therefore, an inadequately designed health app could be inefficient. In addition, the original version of ABACUS demonstrated excellent metric properties and effectiveness in previous studies. As a strength of our study, we highlight the practical implications for developers, clinical practice and researchers. ABACUS-Es provides guidance to apps developers, could be used by healthcare professionals to assess behavior change techniques in apps available in Spanish, and will help guide future research efforts in the development and evaluation of apps to promote health behaviors.

One possible limitation of the study is that the validation was based solely on physical activity apps. Future research could address other behaviors and/or populations to reinforce the results obtained in the present study. Likewise, if effectiveness is observed in interventions where apps are used, subjected to prior analysis of behavior change strategies via the use of ABACUS-Es, this could be regarded as a correlation between the theoretical perspective and the actual results of the intervention, thereby in turn reinforcing the usefulness of ABACUS-Es. Another limitation is that ABACUS-Es cannot be checked alongside another Spanish scale for validity purposes, as no similar scales exist yet. Finally, the metric properties assessed were for Castilian Spanish (Spain), which must be taken into consideration if the version is to be used with other Spanish-speaking populations with a different variety of Spanish, such as Latin American. All those limitations suggest the need of further research assessing the feasibility of ABACUS.

## Conclusions

The results of this study suggest that ABACUS-Es is an instrument with adequate metric properties that can be used to identify behavior change techniques and to measure the potential behavior change of apps. The results of this study show great promise for scale adaptation.

This tool will help health professionals identify behavior change techniques included in apps. It will also consider the best apps based on an empirical evaluation that combines content with quality. Additionally, the tool will also allow developers to design apps.
